# Evidence synthesis in trial design: an example from the neurosciences

**DOI:** 10.1186/1745-6215-14-S1-O6

**Published:** 2013-11-29

**Authors:** Kieren Egan, Hanna Vesterinen, Emily Sena, Malcolm Macleod

**Affiliations:** 1 University of Edinburgh, Edinburgh, UK

## 

The increasing prevalence of Alzheimer's disease (AD) poses a considerable socio-economic challenge. Decades of experimental research have not led to the development of effective disease modifying interventions. A deeper understanding of in vivo research might provide insights to inform future in vivo research and trial design. We therefore performed a systematic review and meta-analysis of interventions tested in transgenic mouse models of AD.

We searched electronically for publications testing interventions in transgenic models of AD. We extracted data for outcome, study characteristics and reported study quality and calculated summary estimates of efficacy using random effects meta-analysis.

We identified 427 publications describing 357 interventions in 55 transgenic models, involving 11, 688 animals in 1774 experiments. Of concern (i) reported study quality was relatively low; fewer than 1 in 5 publications reported the blinded assessment of outcome or random allocation to group and no study reported a sample size calculation; (ii) there were few data for any individual intervention- only 16 interventions had outcomes described in 5 or more publications; and (iii) publication bias analyses suggested 1 in 5 pathological and 1 in 7 neurobehavioural experiments remain unpublished. Of the various pathological outcomes reported, neurodegeneration was the best predictor of neurobehavioural outcome (R^2^= 0.72, p<0.01).

Given these weaknesses in the *in vivo* modelling of AD in transgenic animals and the identified risks of bias, clinical trials based on claims of efficacy in animals should only proceed after it has been shown - through systematic review - that those claims are well founded.

